# Supplementary material for: Prognostic Role of Circulating Tumor Cell Trajectories in Metastatic Colorectal Cancer
Source: Cells. 2023 Apr 16;12(8):1172. doi: 10.3390/cells12081172 (PMC10136568; doi:10.3390/cells12081172)
Supplement: Supplementary file 1 [file cells-12-01172-s001.zip › cells-2288931-supplementary.pdf]

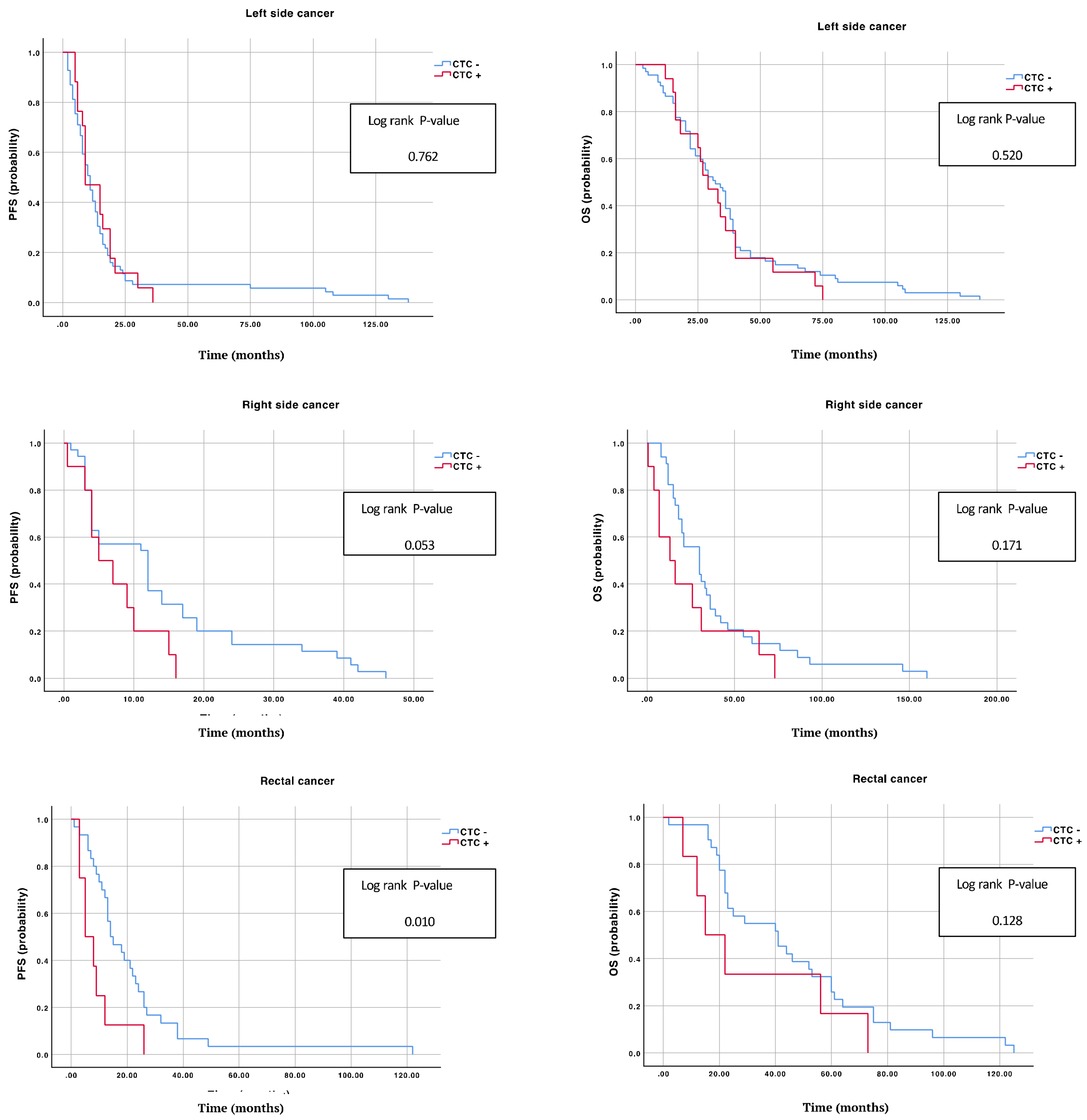

Figure S1. Kaplan-Maier survival curves (probability) for PFS (left panels) and OS (right panels) of different primitive cancer location. Comparison between CTCs negative and CTCs positive at baseline. Top panels: left side cancer (86 patients). Middle panels: right side cancer (45 patients). Bottom panels: rectal cancer (38 patients).

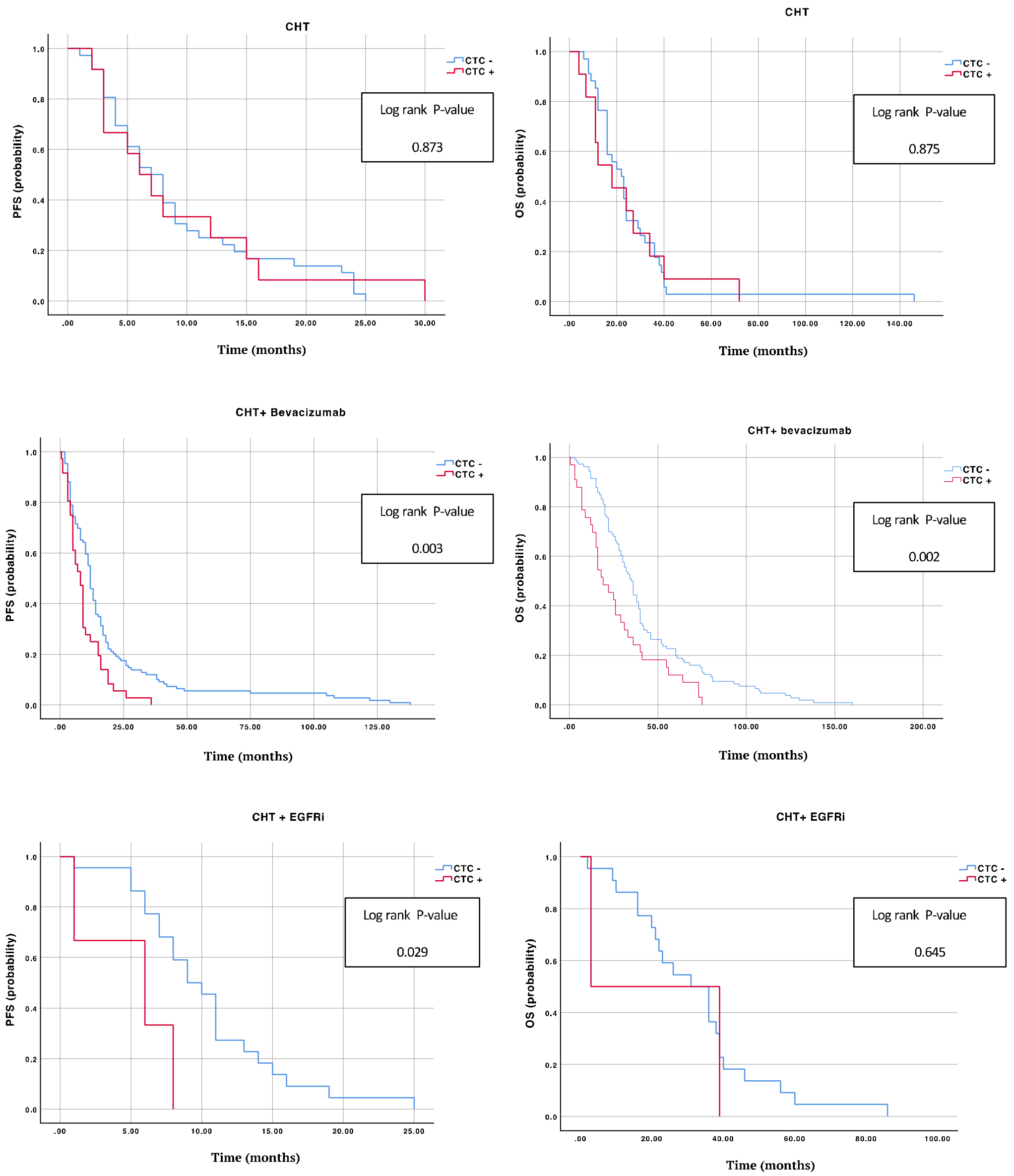

Figure S2. Kaplan-Maier survival curves (probability) for PFS (left panels) and OS (right panels) in different treatments. Comparison between CTCs negative and CTCs positive at baseline. Top panels: CHT therapy (48 patients). Middle panels: CHT + Bevacizumab therapy (145 patients). Bottom panels: CHT + EGFRi therapy (25 patients).
